# Lactate and lactylation in intervertebral disc degeneration

**DOI:** 10.3389/fmolb.2025.1685975

**Published:** 2025-11-18

**Authors:** Yadong Liu, Jihao Yang, Zihui Wang, Jing Fu, Hongpeng Liu, Zekun Lu, Jinwei Dong, Zhong Li, Qiuyue Mao, Chunlan Li, Hui Ma

**Affiliations:** 1 Second Clinical Medical College, Yunnan University of Traditional Chinese Medicine, Kunming, China; 2 Department of Orthopedic, Ninth People’s Hospital, Shanghai Jiao Tong University School of Medicine, Shanghai, China; 3 Department of Emergency, The First Affiliated Hospital of Zhengzhou University, Zhengzhou, China; 4 School of Medicine, Shanghai Jiao Tong University, Shanghai, China; 5 Department of Orthopedic, Affiliated Renhe Hospital of Shanghai University (Renhe Hospital, Baoshan District), Shanghai, China

**Keywords:** intervertebral disc degeneration, lactate, lactylation, metabolism, functions, intervention strategies

## Abstract

The unique hypoxic microenvironment of the intervertebral disc, characterized by its avascularity and restricted nutrient exchange, drives a shift in cellular metabolism towards anaerobic glycolysis. This metabolic adaptation results in the accumulation of significant lactate levels. Increasing evidence indicates that lactate plays a pivotal role in regulating cell differentiation and fate in both physiological and pathological contexts, particularly in complex conditions such as degenerative diseases and cancer. Lactate is not merely a metabolic byproduct; it also modulates cellular signaling pathways and promotes lactylation. In the lactate-enriched microenvironment of the intervertebral disc, understanding the regulatory mechanisms of lactate and lactylation is essential for mitigating intervertebral disc degeneration and improving therapeutic outcomes. Targeting lactate production and transport—particularly through lactate dehydrogenases (LDHs) and monocarboxylate transporters (MCTs)—holds significant therapeutic promise. This review highlights the critical role of lactate in intervertebral disc degeneration progression and discusses potential therapeutic strategies aimed at modulating lactate metabolism to enhance treatment efficacy.

## 1 Introduction

Intervertebral disc degeneration (IDD) is characterized by reduced disc height, diminished hydration, and impaired ability to absorb pressure loads (Kang et al., 2023). Low back pain (LBP), the primary clinical manifestation of IDD, severely impacts patients’ quality of life and generates substantial socioeconomic burdens, contributing to increased healthcare costs and productivity losses (Wu et al., 2020). With the aging global population, the prevalence of IDD is steadily increasing, with projections indicating that by 2025, more than 2.1 billion people will be over the age of 60 (Madhu et al., 2021; Rustenburg et al., 2018; Tonomura et al., 2020). Current management strategies for IDD include both conservative and surgical approaches. Conservative therapies, including physical modalities, pharmacotherapy, and behavioral interventions, offer symptomatic relief but do not halt the progression of degeneration (Xin et al., 2022). Surgical procedures, such as discectomy and spinal fusion, may provide immediate benefits; however, they carry the risk of adjacent segment degeneration, chronic pain, and neurological complications (Mirza and Deyo, 2007). Currently, no clinically validated pharmacological interventions effectively halt or reverse the progression of IDD (Liu et al., 2023). Emerging regenerative strategies, such as stem cell/exosome therapy, gene editing, and molecular targeting, show promising therapeutic potential (Sheyn et al., 2019; Liao et al., 2019; Kamali et al., 2021; Krut et al., 2021). However, significant gaps remain in their clinical translation. Therefore, it is urgent to find effective strategies for treating IDD.

Recent studies have found that lactate plays a significant role in the onset and progression of discogenic degeneration, revealing its multifaceted impact beyond being merely a metabolic byproduct (Grunhagen et al., 2006; Bibby et al., 2005; Wang D. et al., 2021). A key breakthrough in lactate-related research of IDD was the discovery of lactylation, a post-translational modification that bridges metabolism and epigenetics (Zhang et al., 2019). Lactylation is not confined to histones but extends to non-histone proteins, influencing various cellular processes. This modification strengthens the relationship between metabolic states and epigenetic regulation, accelerating the progression of IDD (Sun et al., 2025; Sheng et al., 2024). Lactate-induced lactylation operates through multiple molecular mechanisms and signaling pathways, often interacting with other epigenetic modifications to promote disease progression (Certo et al., 2022; Hu XT. et al., 2024). Notably, lactate and lactylation represent an underexplored therapeutic frontier in IDD. More recently, emerging classical post-translational modifications (PTMs), such as lactylation (Zhang et al., 2019), palmitoylation (Qu et al., 2021), and succinylation (Zhao et al., 2023), have gained attention due to their biological relevance. Therefore, further promotion and consolidation of lactate-related research are urgently needed. This review rationalizes the pathological and physiological effects of lactate, elucidates the molecular mechanisms underlying lactate-driven IDD, and discusses lactate-targeted therapeutic strategies for IDD.

## 2 Intervertebral disc degeneration

**FIGURE 1:**
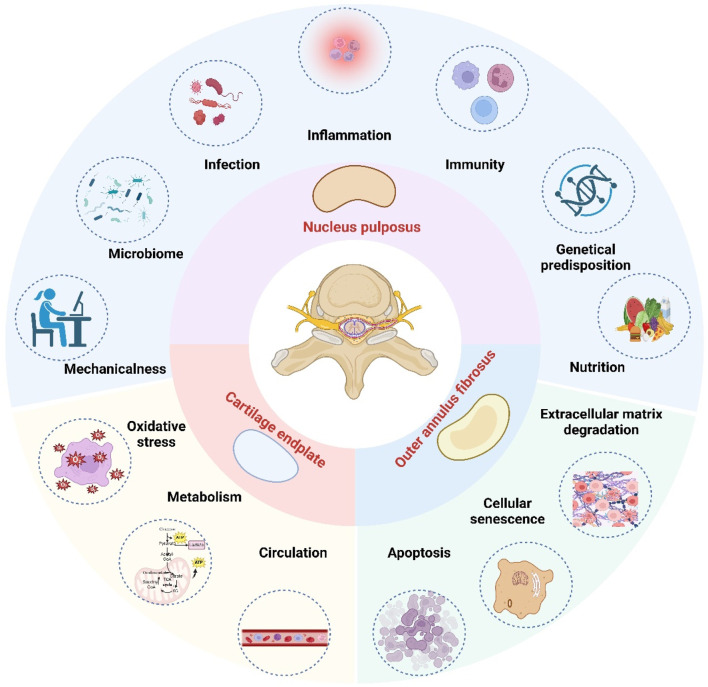
The etiology of intervertebral disc degeneration. In the hypoxic and lactic acidifying environment of the intervertebral disc, the most affected area is the nucleus pulposus, followed by the cartilage endplate and the outer annulus fibrosus. The etiology of intervertebral disc degeneration is multifactorial. Key pathological drivers include oxidative stress, inflammation, apoptosis, cellular senescence, extracellular matrix degradation, nutritional deficiency, metabolic imbalance, genetic predispositions, mechanical stress, autoimmune response, microbial involvement, infection, and circulatory insufficiency. (Figure created with http://www.BioRender.com, accessed on 5 October 2025).

IDD is a highly prevalent spinal disorder that commonly presents as pain and restricted mobility (Dowdell et al., 2017). Anatomically, the disc comprises three distinct components: the outer annulus fibrosus (AF), the inner nucleus pulposus (NP), and the cartilaginous endplates (CEP) (Pan et al., 2018). Current evidence has identified several key pathomechanistic drivers of IDD, including extracellular matrix (ECM) degradation (Roughley et al., 2006; Liang et al., 2022; Wei et al., 2019), oxidative stress (Rahal et al., 2014; Chen X. et al., 2024; Ma et al., 2019; Cheng et al., 2022), cellular senescence (Silwal et al., 2023; Cheng et al., 2025; Shi et al., 2023), and dysregulated autophagy/apoptosis (Madhu et al., 2021; Cheng et al., 2025; Hu et al., 2021; Kang et al., 2020), however, the pathogenesis of IDD is complex and not limited to these factors (Figure 1). The intervertebral disc (IVD) is characterized by an avascular structure and a hypoxic microenvironment, which predominantly relies on glycolysis for energy production. This metabolic reliance leads to the accumulation of substantial amounts of lactate within the IVD (Shen et al., 2022). Only a small portion of this lactate is recycled by nucleus pulposus cells (NPCs) or diffused to surrounding tissues, while efflux mediated by monocarboxylate transporters remains minimal (Bibby et al., 2005; Soukane et al., 2007). The unique structural and metabolic characteristics of the IVD have led to increased focus on lactate and lactylation as important factors in the pathophysiology of IDD.

## 3 Lactate metabolism

### 3.1 History of research on lactate metabolism

The discovery and study of lactic acid (C_3_H_6_O_3_) dates back to the 18th century, when Swedish chemist Carl Wilhelm Scheele first isolated this organic acid from sour milk in 1780 (Kompanje et al., 2007; Mülle et al., 2023). Later, in 1808, renowned chemist Jöns Jacob Berzelius further revealed that athletic muscle tissue also produces lactic acid, establishing its connection to energy metabolism (Dawson et al., 1978). Lactate is the ionized form of lactic acid. Chemically, lactic acid is a three-carbon hydroxycarboxylic acid, which dissociates in aqueous solution to produce lactate ions (C_3_H_5_O_3_-) and hydrogen ions. For a long time, lactate was merely regarded as a byproduct of glycolysis and metabolic waste (Mäki-Arvela et al., 2014). However, the discovery of the Warburg effect and extensive modern research suggest that lactate is not only a crucial energy substrate for skeletal, cardiac, and brain tissues but also acts as a key metabolic signaling molecule in regulating various cellular processes involved in cell fate determination (Grunhagen et al., 2006; Wang and Dang, 2024; Rabinowitz and Enerbäck, 2020; Fendt, 2024); thus, the biological role of lactate has expanded beyond its metabolic functions (Vander Heiden et al., 2009). As research into lactate and lactylation in IDD continues to grow, strategies targeting lactate and lactylation have gained considerable interest as potential therapeutic interventions for IDD.

### 3.2 Lactate production

During high energy demand, glucose is catabolized through the glycolytic pathway, ultimately generating pyruvate. Lactate dehydrogenase (LDH) plays a key catalytic role in converting most of the pyruvate to lactate (Sharma et al., 2022). The physiological significance of lactate production lies primarily in maintaining the NAD^+^ pool required for glycolysis through the coupled oxidation of NADH to NAD^+^ (Cluntun et al., 2021; Arce-Molina et al., 2020), and in sustaining the reaction catalyzed by 3-phosphoglycerol aldehyde dehydrogenase, thereby maintaining glycolytic flux and ATP production (Henderson et al., 2007; Mitchell, 1961). To regulate lactate metabolism, the body has established intricate metabolic regulatory mechanisms. The pyruvate dehydrogenase (PDH) complex catalyzes the irreversible conversion of pyruvate to acetyl-CoA, which then enters the tricarboxylic acid (TCA) cycle for complete oxidation (Breda et al., 2019). Alternatively, excess lactate can be converted back into glucose via gluconeogenesis (Andreucci et al., 2020; H et al., 2012). This dynamic equilibrium mechanism satisfies energy demands while preventing metabolic acidosis due to excessive lactate accumulation.

**FIGURE 2:**
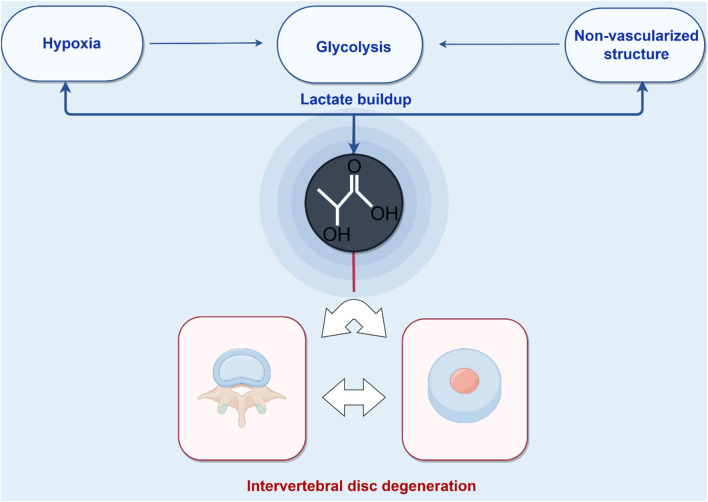
Pathological lactate accumulation in the intervertebral disc microenvironment. Energy homeostasis in the avascular nucleus pulposus (NP) critically depends on glycolytic flux. Lactate, a key byproduct of glycolysis, accumulates and drives lysine lactylation on histone and non-histone proteins, establishing a feed-forward loop that accelerates intervertebral disc degeneration (IDD). (Figure created with http://www.home-for-researchers.com, accessed on 5 October 2025).

The avascular, hypoxic microenvironment of the IVD forces reliance on anaerobic glycolysis, particularly in NPCs, as the primary energy-producing pathway. This metabolic adaptation leads to significant lactate production within the IVD (Bibby et al., 2005; Soukane et al., 2007) (Figure 2). Physiological homeostasis is maintained through the dynamic regulation of lactate flux, including its synthesis, transmembrane transport, and clearance mechanisms (Silagi et al., 2020; Silagi et al., 2021). However, in degenerative conditions, lactate concentrations increase significantly, with the highest accumulation observed in the central NP regions of degenerated discs (Shi et al., 2019; Malandrino et al., 2011). This metabolic disruption arises from compromised nutrient and metabolite diffusion through adjacent capillary networks (Shen et al., 2022; Yang et al., 2020), resulting in NP lactate levels that are 8–10 times higher than those in circulating plasma (Shen et al., 2022; Silagi et al., 2018). Pathologically elevated lactate levels directly inhibit ECM biosynthesis and promote NPCs apoptosis (Shen et al., 2022; Shi et al., 2019), accelerating IVD structural degradation and exacerbating discogenic pain pathways (Shen et al., 2022; Keshari et al., 2008; Byvaltsev et al., 2018). In addition, this metabolic disturbance creates an acidic microenvironment that promotes ECM acidification, inhibits proteoglycan biosynthesis, and activates pro-inflammatory cascades, all of which accelerate the progression of IDD (Shi et al., 2019; Ma et al., 2024; Zhao et al., 2021). Emerging evidence suggests that lactate accumulation and its derivative lactylation play central roles in driving IDD pathogenesis (Shen et al., 2022). Therefore, understanding the mechanistic role of lactylation in IVD degeneration is critical for advancing the knowledge of NP cell pathophysiology and for developing targeted therapeutic strategies against IDD.

### 3.3 Lactate transport and shuttle

Lactate transport within cells is primarily mediated by the monocarboxylate transporter (MCT) family, including isoforms such as MCT1 and MCT4. These transporters facilitate transmembrane lactate transport through the H^+^/lactate cotransport mechanism, with transport direction determined by the transmembrane proton and lactate concentration gradients (Slomiany et al., 2009; Fang et al., 2023; Fan M. et al., 2023). MCT-mediated lactate efflux also removes protons, maintaining intracellular pH homeostasis while acidifying the extracellular microenvironment (Fan M. et al., 2023; Chen et al., 2025). Among the MCT family, MCT1 is a basally expressed transporter regulated by c-Myc, widely distributed across various cell types, and responsible for the transport of lactate and pyruvate (Doherty et al., 2014). In contrast, MCT4, a hypoxia-inducible and efficient lactate transporter, is particularly expressed in glycolytically active tissues, such as white muscle fibers and tumor cells (Fang et al., 2023; Singh et al., 2023). Notably, the NP of the IVD, a glycolysis-dependent tissue, upregulates MCT expression during degeneration, promoting the formation of an acidic microenvironment (Wang CY. et al., 2022). Aberrant expression of monocarboxylate transporters (MCT1/4) exacerbates lactate retention, creating a self-perpetuating pathological loop (Wang D. et al., 2021). This acidic microenvironment, in turn, maintains high glycolytic activity through a positive feedback mechanism, significantly influencing the survival and function of NPCs (Wang CY. et al., 2022). These findings provide new insights into the molecular mechanisms of IDD.

Since its introduction in 1985, the lactate shuttle theory has become a fundamental concept in metabolic research, continuously validated and expanded. The theory systematically describes the dynamic transport of lactate, both as a glycolytic end product and as a substrate for oxidative respiration, across cells, tissues, and organs. Specifically, the lactate shuttle not only elucidates the molecular mechanisms of lactate transmembrane transport but also highlights its critical role as a metabolic bridge connecting anaerobic glycolysis with aerobic respiration (Brooks, 2018; Li et al., 2022; Wang Y. et al., 2022). Notably, this metabolic connection is maintained even under normoxic conditions (Brooks, 2009; Halestra and p, 2013). From a physiological perspective, the lactate shuttle serves three key functions: first, lactate acts as an important energy carrier for the organism; second, it is a key precursor for gluconeogenesis; and third, lactate functions as an autocrine, paracrine, and endocrine-like signaling molecule.

A notable example is the muscle-hepatic Cori cycle, which accomplishes three major physiological functions: it effectively prevents muscle lactic acidosis during exercise, sustains muscle ATP supply, and provides a more significant gluconeogenic substrate than dietary sources by converting muscle-generated lactate into glucose via hepatic gluconeogenesis, which is then resupplied to the muscle (Manoj et al., 2022; Ku et al., 2024; Soeters et al., 2021). Additionally, lactate can be transported between cells in a non-channel-dependent manner, expanding our understanding of lactate’s metabolic networks beyond the classical MCT transporter protein-mediated pathway (Chen et al., 2025; Py et al., 2005). Finally, research has demonstrated lactate-dependent metabolic symbiosis within the disc, where lactate produced by hypoxic, glycolytic NP cells is oxidatively phosphorylated by oxygenated AF cells for energy and substrate production. This paradigm shift challenges the traditional view of disc lactate as a waste product, recognizing it instead as a valuable biofuel (Wang D. et al., 2021).

### 3.4 Biological functions of lactate

#### 3.4.1 Redox steady-state-buffer

Lactate, as a key metabolic intermediate, plays a pivotal role in cellular redox signaling. Its metabolic processes maintain electron homeostasis through specific pathways, including the conversion of NADH to NAD^+^ and H^+^ and the lactate-pyruvate interconversion mediated by LDH (Titov et al., 2016). These reactions involve the oxidation of reduced coenzymes (NAD^+^/NADP^+^) to generate electrons via the mitochondrial respiratory chain or the lactic acid fermentation pathway, thereby regulating redox balance. Notably, lactate also serves as a regulator of oxidative phosphorylation (OXPHOS) and is involved in maintaining intracellular redox homeostasis (Ying, 2008).

#### 3.4.2 Circulating carbohydrate fuel of the TCA cycle

Recent studies show that lactate can also serve as a major carbon source for the TCA cycle (Hui et al., 2020). Using the ^13^C isotope tracer technique, studies revealed that (1) the circulating turnover flux of lactate in mammals is significantly higher than that of glucose, reaching 1.1 and 2.5 times the turnover flux of glucose in fed and fasted states, respectively; (2) while glucose is the primary precursor of lactate, other metabolic pathways also contribute to lactate production; (3) ^13^C labeling experiments confirmed that lactate is widely involved in TCA cycle metabolism in various tissues and organs (Hui et al., 2017). Additionally, lactate has been shown to function as an important energy carrier for the central nervous system, both by maintaining the energy homeostasis of preopiomelanocortin (POMC) neurons and directly supporting the energy demands of excitatory neural activities in the brain. These findings have fundamentally altered our understanding of lactate’s metabolic role, positioning it as a central hub in energy metabolism (Dienel, 2019; Gómez-Valadés et al., 2021).

## 4 The multifaceted functions of lactate in intervertebral disc degeneration

### 4.1 Decreased proteoglycan content

An acidic environment significantly inhibits proteoglycan biosynthesis in cartilage tissue. To explore this mechanism, researchers systematically evaluated the effects of lactate concentration and pH changes on IVD matrix synthesis in experimental samples, including bovine caudal IVDs and human IVD tissues obtained from percutaneous nucleotomy. The rate of proteoglycan synthesis was found to be highly sensitive to extracellular pH, with optimal synthesis efficiency occurring within a range close to physiological pH, as measured by ^35^S-sulfate and ^3^H-proline isotope labeling assays (Ohshima and Urban, 1992). Notably, factors that promote lactate accumulation (such as hypoxia, smoking, or mechanical vibration) can inhibit proteoglycan synthesis by lowering local pH (Ohshima and Urban, 1992). This ongoing impairment in synthesis can ultimately lead to a progressive reduction in proteoglycan content within the disc matrix, accelerating the process of IDD. These findings provide essential experimental evidence for understanding the pathological links between mechanical stress, metabolic disorders, and IDD.

Under physiological conditions, the IVD environment typically maintains a relatively neutral pH (pH 7.2-7) (Urban, 2002). However, when this homeostasis shifts toward an acidic environment, the normal metabolic functions of the disc are significantly impaired. Urban and his team have demonstrated through a series of experiments that a decrease in pH triggers a series of pathological changes: on one hand, it promotes the upregulation of ECM-degrading enzymes, such as matrix metalloproteinases (MMPs), and on the other hand, it inhibits the synthesis of key matrix components, such as proteoglycans (Bibby et al., 2005; Bibby et al., 2002; Urban and Mcmullin, 1988). This metabolic disturbance not only disrupts the structural integrity of the IVD but also negatively impacts its biomechanical properties, which may ultimately lead to abnormal disc function and contribute to chronic LBP. These findings provide important experimental support for understanding the causal relationship between the acidic microenvironment and IDD.

### 4.2 Inflammation and apoptosis

Lactate plays a dual role in tissue repair and regeneration (Rabinowitz and Enerbäck, 2020). At physiological concentrations, lactate acts as an important metabolic signaling molecule, promoting wound healing by regulating cellular signaling, stimulating neovascularization, and enhancing fibroplasia, among other mechanisms (Zhang et al., 2019; Brooks, 2018). However, pathological lactate accumulation triggers a range of harmful effects, including excessive recruitment of inflammatory cells, mitochondrial dysfunction, and increased production of reactive oxygen species (ROS), which ultimately lead to the death of tissue-resident cells and hinder regenerative processes (Ivashkiv, 2020; Pucino et al., 2017). In the pathological context of IDD, lactate levels in the NP tissue can be 8 to 10 times higher than in the surrounding plasma due to impaired diffusion of metabolites and nutrients through adjacent capillaries (Yang et al., 2020; Silagi et al., 2018). This abnormal lactate buildup not only inhibits ECM synthesis but also promotes NP cell apoptosis (Shi et al., 2019), leading to structural damage in the IVD and discogenic pain (Shen et al., 2022; Keshari et al., 2008). Additionally, 2 mM lactate promotes NP cell proliferation, while 6 mM lactate slightly inhibits proliferation and induces autophagy and apoptosis in NP cells (Wu et al., 2014).

Notably, lactate gradually accumulates in the degenerating region, from the periphery of the AF toward the center of the NP, forming a gradient distribution along with a decreasing pH. This acid-base gradient change activates multiple inflammatory pathways, further intensifying the local inflammatory response (Ma et al., 2024; Horner et al., 2002; Zhang et al., 2024a). In this hyper-inflammatory environment, the regenerative capacity of NP cells is significantly impaired, leading to a severe imbalance between ECM synthesis and degradation. This imbalance ultimately triggers disc herniation and painful symptoms (Ma et al., 2024; Liu et al., 2017). Moreover, the inflammatory microenvironment in the degenerated disc accelerates disease progression through a dual mechanism: it directly disrupts the metabolic function of NP cells, worsening ECM metabolic disorders (Gaudet and Popovich, 2014; Li et al., 2023), and it triggers a cascade effect that recruits more inflammatory cells, further amplifying the inflammatory response (Ma et al., 2024; Gao et al., 2023). This vicious cycle leads to irreversible pathological changes.

### 4.3 Pyroptosis and signaling

Unlike conventional cell death, pyroptosis is a form of programmed necrosis with pro-inflammatory properties. It is characterized by cellular swelling until membrane rupture, resulting in the leakage of intracellular contents and triggering a more intense inflammatory response (Broz, 2025). In recent years, inflammation-mediated cellular pyroptosis has been recognized as a key player in IDD (Wang et al., 2024). Extracellular lactate not only promotes the activation of NLRP3 inflammasomes, leading to degeneration of the ECM in the NP, but also significantly enhances the inflammatory response and increases the level of cellular pyroptosis in NP tissues (Okajima, 2013; Chen et al., 2022; Song et al., 2025). At the molecular level, the NLRP3 inflammasome serves as the central molecular machinery mediating typical cellular juxtaposition (Coll et al., 2022; Hsu et al., 2021). Atypical inflammasomes, composed primarily of caspase-4/5/11, specifically recognize lipopolysaccharides in the cytoplasm and induce cellular pyroptosis by cleaving the downstream effector protein GSDMD (Song et al., 2025; Miao et al., 2023). In addition, inflammatory cytokines accelerate the IDD process by activating multiple signaling pathways, including NF-κB, MAPK, and PI3K/Akt, triggering intracellular inflammatory cascades and pyroptosis (Song et al., 2025; Liu et al., 2018; Zhou et al., 2024; Guo et al., 2023). These findings provide a new theoretical basis for understanding the lactate-NLRP3-pyroptosis axis in IDD.

Acid-sensing ion channels (ASICs) are important members of the proton-gated ion channel family, consisting of six isoforms (ASIC1a, 1b, 2a, 2b, 3, and 4) that can be activated by ligands such as extracellular acidosis, lactate, and arachidonic acid (Omerbašić et al., 2015; Zhou et al., 2016). These channels are widely expressed in various mammalian tissues, including the nervous system, articular chondrocytes, and cells of the musculoskeletal system and IVD (Song et al., 2025; Ruan et al., 2021; Gilbert et al., 2016). The expression levels of ASIC1, ASIC2, and ASIC3 are significantly upregulated in degenerative NP cells (Song et al., 2025). Extracellular lactate not only promotes the activation of NLRP3 inflammasomes through ASIC activation, leading to degeneration of the NP ECM, but also significantly enhances the inflammatory response and the level of cellular pyroptosis in NP tissues (Song et al., 2025). This discovery provides a novel molecular perspective for understanding the pathological mechanisms of the acidic microenvironment in IDD.

### 4.4 Metabolic regulation

In severely degenerated human NP and senescent SD rat NP tissues, decreased glutamine levels were accompanied by lactate accumulation and enhanced protein lactylation (Zhang et al., 2024b). Mechanistic studies showed that exogenous glutamine supplementation could rectify this imbalance by: (1) reducing lactate production by inhibiting the glycolytic pathway; (2) downregulating the lactylation of the AMPKα subunit; (3) promoting the activation of AMPKα phosphorylation. Further experiments confirmed that glutamine supplementation reduces the senescence phenotype of NP cells, enhances cellular autophagy activity, and promotes ECM synthesis through these dual regulatory mechanisms (Zhang et al., 2024b). This study reveals the central role of glutamine metabolic regulation in maintaining NP cells homeostasis through dual mechanisms: inhibiting glycolysis and lactate production, and activating AMPK phosphorylation signaling, thereby providing a novel theoretical basis for metabolic-targeted strategies against IDD.

### 4.5 Oxidative stress and cellular senescence

High lactate concentrations can accelerate tissue degeneration through various molecular mechanisms. Lactate interacts with Akt proteins, inducing cellular senescence and oxidative stress by modulating the Akt/p21/p27/cyclin D1 and Akt/Nrf2/HO-1 signaling pathways (Zhang et al., 2024a). This effect has been clearly demonstrated in NPCs. Notably, the association between lactate accumulation and senescence has been observed across different species, with the *Drosophila* model showing a correlation between lactate levels and the senescence process (Iatsenko et al., 2018). In the nervous system, elevated lactate levels serve as a key biomarker of brain aging (Ross et al., 2010), and their accumulation promotes the progression of neurodegenerative diseases such as Alzheimer’s disease (Wang Q. et al., 2022; Mullins et al., 2018). Moreover, lactate induces tissue damage by triggering oxidative stress during peripheral nerve regeneration disorders and arterial calcification (Zhang et al., 2024a; Zhu et al., 2019; Jia et al., 2021). Collectively, these findings underscore the critical role of lactate metabolism disorders in various degenerative diseases.

In addition to the roles of lactate mentioned above,​​ recent study has revealed that in myelofibrosis (MF), the abnormal accumulation of lactate can remodel the bone marrow microenvironment, driving the fibrotic progression of the disease by inducing an immunosuppressive state and promoting collagen deposition (Spampinato et al., 2025). This study clearly identifies lactate as a core regulator of immune escape and fibrotic transformation in MF, and proposes targeting the lactate transporter MCT1 as an innovative anti-fibrotic strategy (Spampinato et al., 2025). This provides a highly valuable parallel perspective for understanding the potential function of lactate in IDD: NPCs during degeneration also exhibit significantly activated glycolysis and substantial lactate accumulation (Xu et al., 2025; Peng et al., 2024). Could the large amount of lactate produced by NPCs under high mechanical load and hypoxic conditions in degenerated discs similarly drive the imbalance between destruction and repair of the annulus fibrosus through analogous mechanisms? Furthermore, the MCT1 blockade strategy proposed in MF research also offers important insights for exploring new therapeutic targets to delay or reverse disc fibrosis.

## 5 Lactylation: a bridge between metabolism and epigenetics

**FIGURE 3:**
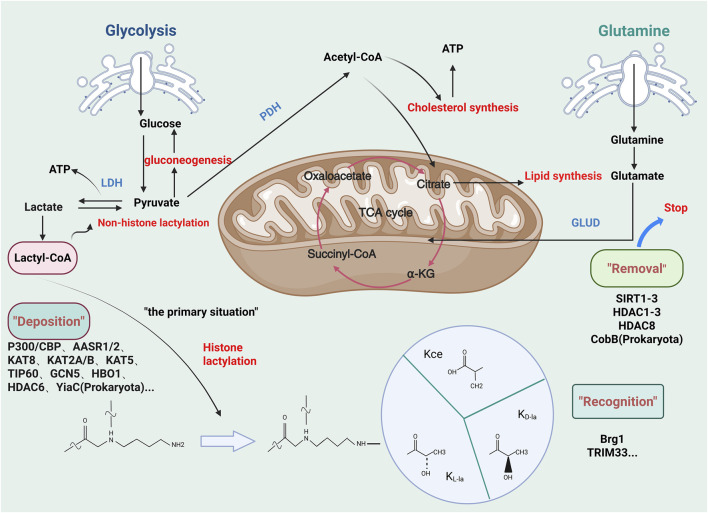
Regulatory mechanisms involved in lactylation. Glycolysis generates pyruvate, which leads to lactate accumulation in intervertebral disc degeneration (IDD). This lactate pool promotes lysine lactylation (KL-la), a novel post-translational modification mediated by L-lactate. Related isomers, including N-ε-(carboxyethyl)-lysine (Kce) and D-lactate-lysine (KD-la), share similar molecular structures but differ in stereochemistry. Lactylation mechanisms can be categorized into enzymatic and non-enzymatic pathways based on precursor specificity. The enzymatic KL-la cascade, the most studied pathway, operates through a tripartite enzymatic machinery: “Writer” (deposition), “Reader” (recognition), and “Eraser” (removal) proteins. (Figure created at http://www.BioRender.com on 7 October 2025).

Histone lactylation was first documented in 2019 by Zhang et al. (Zhang et al., 2019) (Figure 3). This PTM involves the covalent attachment of lactate to lysine residues via amide bonds, forming lactylated lysine (Kla). Notably, lactylation redefines lactate—previously considered a metabolic byproduct—as an epigenetic regulator, positioning it as an emerging research frontier (Zhang et al., 2019). Biochemically, lactylation is dependent on lactate’s α-hydroxycarboxylic acid structure (Chen H. et al., 2024; Jing et al., 2025), and it is catalyzed by lactyltransferase enzymes (such as p300) through lactyl-CoA intermediates (Zhang et al., 2019). We summarize inhibitors of lactylation and enzymes associated with lactylation (Tables 1, 2). The levels of lactylation increase under conditions of elevated intracellular lactate concentrations, particularly during hypoxia or hyperglycemia, thus linking metabolic flux directly to gene regulation (Zhang et al., 2019). Notably, site-specific modifications, such as histone H3K18la, drive pro-inflammatory gene transcription, highlighting lactylation’s critical role in immunometabolic reprogramming (Piao et al., 2025). This paradigm establishes a novel metabolic-epigenetic axis, challenging the classical view of metabolites as merely energy substrates or signaling molecules.

**TABLE 1:** Inhibitors of lactylation.

Name	Targets	Mechanism	Control method	Status	References
Gne-140	LDHA/B	Inhibition of lactate dehydrogenase	Indirect regulation	*In vivo*/*vitro*	Boudreau et al. (2016)
GSK-2837808A	LDHA	Inhibition of lactate dehydrogenase	Indirect regulation	*In vitro*	Yin et al. (2024), Dong et al. (2022a)
Stiripentol	LDHA	Blocking lactate production in gastric cancer cells and inhibiting lactylation of NBS1 K388	Indirect regulation	*In vivo*/*vitro*	Che et al. (2022)
Oxamate	LDHA	Competitive inhibition of LDH activity	Indirect regulation	*In vivo*/*vitro*	Li et al. (2024a)
FX11	LDHA	Targeting LDHA subunits to inhibit lactate production in tumor cells	Indirect regulation	*In vitro*	Tong et al. (2024)
A485	P300/CBP	Inhibition of histone lactyltransferase activity (P300/CBP)	Indirect regulation	*In vivo*/*vitro*	Jaschke et al. (2024), Lasko et al. (2017)
C646	P300/CBP	Inhibition of histone lactyltransferase activity (P300/CBP)	Indirect regulation	*In vivo*/*vitro*	Lasko et al. (2017)
MG149	TIP60、MOF	Inhibits histone lactyltransferase activity (TIP60, MOF)	Indirect regulation	*In vivo*/*vitro*	Wang et al. (2022d)
AZD-3965	MCT1	Inhibition of the lactate transporter MCT1	Indirect regulation	*In vivo*	Halford et al. (2023)
SR13800	MCT1	Inhibition of the lactate transporter MCT1	Indirect regulation	*In vivo*/*vitro*	Chatterjee et al. (2023), Khan et al. (2020)
α-CHCA	MCT1	Inhibition of the lactate transporter MCT1	Indirect regulation	*In vivo*/*vitro*	Chatterjee et al. (2023), Wan et al. (2021)
DCA	PDK	Activating pyruvate dehydrogenase and reducing lactate production	Indirect regulation	*In vitro*	Nguyen et al. (2025)
2-DG	Hexokinase (HK)	Inhibiting glycolysis and reducing lactate production	Indirect regulation	*In vivo*/*vitro*	Cheng et al. (2021)
AT-101	Bcl-2 family proteins	Inhibiting glycolysis and lactate production	indirect regulation	*In vivo*/*vitro*	Moretti et al. (2010)
Quercetin	MCT4	Inhibiting LDH activity and reducing intracellular lactate levels	Indirect regulation	*In vivo*/*vitro*	Zhu et al. (2024)
Oxymatrine	TLR2	Indirect regulation of lactylation through inflammatory signaling pathways	Indirect regulation	*In vivo*/*vitro*	Ni et al. (2025)
Aloe-emodin	P4HB	Blocking P4HB lactylation	Indirect regulation	*In vivo*/*vitro*	Ouyang et al. (2024)

Abbreviations: LDHA/B: lactate dehydrogenase A/B; P300/CBP: histone lactyltransferases; TiP60: tat-interactive protein 60; MOF: males absent on the First; MCT1/2: monocarboxylate transporter1/2; DCA: dichloroacetate; 2-DG: 2-deoxyglucose; TLR2: toll-like receptor 2; P4HB: prolyl 4-hydroxylation subunit beta.

**TABLE 2:** Enzymes involved in modifications mediated by lactylation.

Function	Gene	Role	References
Writer	AARS1/2	Lactate sensor; lactate transferase (LAT); utilizing lactate as a substrate	Li et al. (2024b)
Writer	EP300	Mediating the lactylation of lysine residues on target proteins	Iozzo et al. (2025)
Writer	KAT2A		Zhu et al. (2025)
Writer	KAT2B		Lu et al. (2025a)
Writer	KAT5		Zou et al. (2025)
Writer	KAT7		Wang et al. (2025)
Writer	KAT8		Xie et al. (2024)
Writer	yiaC		Dong et al. (2022b)
Writer	TIP60		Chen et al. (2024b)
Writer	GCN5		Wu et al. (2023)
Writer	HBO1		Niu et al. (2024)
Writer	HDAC6		Sun et al. (2024)
Reader	Brg1	Histone lactylation readers	Hu et al. (2024b)
Reader	TRIM33		Nu et al. (2024)
Eraser	HDAC1	Facilitating the hydrolysis of lactyl ation moieties from modification sites, thus reversing lactylation	Moreno-Yruela et al. (2022)
Eraser	HDAC2		
Eraser	HDAC3		
Eraser	HDAC8		Sang et al. (2024)
Eraser	cobB		Xu et al. (2019)
Eraser	SIRT1		Du et al. (2024)
Eraser	SIRT3		
Eraser	SIRT2		Huang et al. (2024)

Abbreviations: AARS, aminoacyl-tRNA, synthetases; KAT, histone acetyltransferase; HDAC, histone deacetylase; GCN5, general control nonderepressible; Brg1, brahma-related gene 1; TRMI33, tripartite motif containing 33.

Lactylation facilitates the crosstalk between metabolism and epigenetics (Zhang et al., 2019; Fan H. et al., 2023; Wang et al., 2023; Izzo and Wellen, 2019). Recent single-cell RNA sequencing analyses demonstrate that during the progression of IDD, NPCs exhibit enhanced glycolytic activity, with the resulting lactate excess leading to NPC dysfunction through the activation of the ferroptosis pathway (Sun et al., 2025). At the molecular level, lactate regulates acyl-CoA synthetase long-chain family member 4 (ACSL4) through a dual mechanism: it promotes lactylation at the histone H3K18 site, which upregulates ACSL4 transcription, and it directly enhances ACSL4 protein lactylation at the K412 site (Sun et al., 2025). Furthermore, lactate exacerbates ACSL4 lactylation by inhibiting Sirtuin-3 (SIRT3) expression (Sun et al., 2025). These findings establish a new theoretical framework for understanding lactate-mediated epigenetic regulation in IDD.

## 6 Clinical applications and intervention strategies

Lactate homeostasis imbalance plays a pivotal role in the pathogenesis of IDD and could serve as a potential therapeutic target. In terms of molecular mechanisms, lactate metabolism is regulated by two key proteins: LDH, which catalyzes the conversion of pyruvate to lactate, and inhibition of LDH activity effectively reduces lactate production (Sharma et al., 2022; Chen et al., 2025; An et al., 2023). MCTs are responsible for lactate transmembrane transport, and targeted interventions can block lactate release (Py et al., 2005). Importantly, modulating lactate levels could serve as an independent therapeutic strategy, potentially synergizing with conventional adjunctive therapies such as physical therapy and rehabilitation exercises, providing new avenues for the comprehensive treatment of IDD.

### 6.1 Detection of lactate

Advanced molecular and imaging techniques offer valuable insights into the mechanisms and functional importance of fluctuating tissue lactate levels during the progression of IDD. Magnetic resonance spectroscopy (MRS) and T2 relaxation time (T2r) measurement techniques can act as sensitive imaging biomarkers for early IDD, enabling early diagnosis through the detection of subtle changes in metabolic activity within the disc matrix (Bhari Thippeswamy et al., 2025). In a study involving 236 normal volunteer discs and 215 disc samples from patients with LBP, the molecular profiles of several metabolites, including lactate, were obtained using MRS technology, while disc hydration and collagen content were assessed via T2r measurements (Bhari Thippeswamy et al., 2025). Notably, results indicated that even within discs of the same Pfirrmann classification (PF1), metabolite concentrations such as lactate differed significantly between normal volunteers and patients with LBP (Bhari Thippeswamy et al., 2025). Additionally, the combination of a clinical 3T magnetic resonance (MR) scanner and short-echo water suppression point-resolved spectroscopy (PRESS) technology enables noninvasive, reproducible quantitative detection of metabolic changes associated with IDD (Zuo et al., 2009). The study systematically analyzed bovine IVDs with papain-induced degeneration (N = 17) and human cadaveric IVDs (N = 27) based on Pfirrmann grading of T2-weighted images. Using the 1H PRESS technique, the researchers successfully quantified metabolite concentrations in the carbohydrate zone (Carb), choline head group (Cho), N-acetyl zone (N-acetyl), and lipid and lactate complex (Lac + Lip), and compared metabolic differences between samples with varying levels of degeneration (Zuo et al., 2009). These innovative methods provide a reliable clinical tool for the early diagnosis and metabolic assessment of IDD.

As an emerging area of research in epigenetic modifications, the study of protein lactylation (especially non-histone lactylation) is still in its early stages, underscoring the urgent need for the development of highly sensitive and specific detection techniques. Wan’s research team made a pioneering discovery that during tandem mass spectrometry, lactoyl-lysine (K-lac) forms characteristic cyclic immonium (CycIm) ions, providing a breakthrough for the precise identification of protein lactylation (Wan et al., 2022). The team systematically validated the sensitivity and specificity of this method using affinity-enriched lactylproteome proteomics combined with in-depth informatics evaluation of non-lactylated spectral databases (Wan et al., 2022). Meanwhile, Sun’s group developed an innovative chemical probe, sodium (S)-2-hydroxypent-4-ynoate (YnLac), which can specifically integrate fluorescent or affinity tags into lactated proteins via metabolic labeling strategies. This enables fluorescence visualization of lactylation for detection and proteomics analysis. These technological advances have established a crucial methodological foundation for the in-depth analysis of the biological functions of lactylation (Sun et al., 2022). In conclusion, there is an urgent need to develop high-throughput, high-resolution detection systems to systematically uncover the dynamic regulation of lactate/lactylation in different disc cell populations and their molecular mechanisms in the degeneration process. This will form the theoretical foundation for the development of precise therapeutic strategies targeting lactate metabolism.

Future studies could further integrate single-cell multi-omics technologies to provide finer resolution for studies on disc lactate/lactylation. By combining single-cell transcriptome sequencing (scRNA-seq) with lactate/lactylation proteomic analysis, it will be possible to precisely distinguish the lactate/lactylation characteristics of different cell subgroups, such as NP and AF cells. Additionally, cell-type-specific lactate/lactylation maps can be established, allowing the identification of key gene networks significantly correlated with lactate/lactylation levels in specific cell populations. This multimodal analysis will not only elucidate the metabolic heterogeneity in IDD but also provide novel molecular markers for disease classification and prognostic assessment.

### 6.2 Targeting LDHs

Studies have shown that LDHA inhibitors can effectively reduce lactate levels by blocking the final step of glycolysis, thereby inhibiting tumor progression (Le et al., 2010). Furthermore, *in vitro* and *in vivo* studies have demonstrated that the inhibition of glycolytic activity resulting from LDHA knockdown confers antitumor effects in pancreatic ductal adenocarcinoma (PDAC) (Li F. et al., 2024). However, another study found that high expression of SIRT1 correlates with elevated LDHA expression, suggesting that increased glycolysis generates more ATP to support physiological activities, thereby preventing the negative effects of incomplete OXPHOS on NP cells (Wu et al., 2021). These findings prompt us to consider: Could targeting LDH to inhibit the glycolytic process be a viable approach for treating IDD? Although research on targeting LDH for IDD treatment remains scarce, LDH is undoubtedly a highly promising therapeutic target, providing a crucial therapeutic strategy for delaying the degeneration of nucleus pulposus cells in intervertebral discs.

### 6.3 Targeting MCTs

Recent study has found that inhibition of lactate influx by the monocarboxylate transporter (MCT)-1 inhibitor, AZD3965, reversed the effect of lactate on GAG accumulation and MMP3 expression and further improved NP cell degeneration in the NPD model (Wang CY. et al., 2022). Currently, MCT inhibitors are primarily used in tumor therapy, with first-generation broad-spectrum inhibitors like α-cyano-4-hydroxycinnamic acid (CHC) (Miranda-Gonçalves et al., 2013; Amorim et al., 2015) and photothialdehydes benzenesulfonates (Poole and Halestrap, 1990), as well as second-generation inhibitors such as AR-C155858 (Andersen et al., 2016; Lopez et al., 2023; Richiardone et al., 2024), BAY-8002 (Wang N. et al., 2021; Vera et al., 2024), and SR13800 (Chatterjee et al., 2023; Khan et al., 2020; Padilla et al., 2022). These drugs have shown promising effects in regulating tumor metabolism, but their potential for treating IDD needs systematic evaluation. Despite the rapid progress in developing MCT inhibitors, some issues also need attention. These inhibitors may interfere with normal cellular energy metabolism, potentially causing severe nonspecific toxic responses. Notably, a recent innovative strategy in oncology therapy—blocking the pathway linking metabolic reprogramming and proteomic remodeling in tumor cells by inhibiting aminoacyl-tRNA synthetase 1 (AARS1) (Li H. et al., 2024; Zong et al., 2024; Li et al., 2025)—offers a new approach for treating IDD. However, there is a gap in research on AARS1 and its cognate protein AARS2 inhibitors in the context of IDD.

This opens a new research direction for developing therapeutic strategies targeting lactate metabolism in IDD. Future studies could build on oncology research to explore the potential application of these targeted drugs in IDD treatment, with special attention to their selectivity and safety.

### 6.4 Other intervention strategies

Recent advances in nanomaterials science have introduced new possibilities for treating metabolic diseases. A recent study reported a microfluidics-based nanoenzyme functionalized delivery system (MS@MCL), which involves grafting manganese dioxide (MnO_2_)-lactate oxidase (LOX) composite nanoenzymes onto the surface of hyaluronate methacrylate (HAMA) microspheres via chemical bonding (Shen et al., 2022). This system offers several significant advantages: (1) the uniform porous structure created through microfluidics significantly enhances encapsulation efficiency and injection performance; (2) chemical grafting via amide reaction ensures localized enzyme enrichment and activity enhancement; and (3) sustained oxygen-promoted lactate consumption capacity, along with an extended *in vivo* half-life. The study demonstrated for the first time that nanoenzyme-functionalized injections can significantly promote the regenerative repair of ischemic tissues by modulating the local lactate microenvironment, offering a novel approach to the treatment of IDD (Shen et al., 2022). However, to achieve a major breakthrough in lactate-targeted therapeutics, several key scientific challenges must still be addressed: first, the molecular regulatory mechanisms of lactate/lactylation and their dynamic changes need to be thoroughly elucidated; second, the interaction network of lactate metabolism-related signaling pathways, along with their spatial and temporal specificity, must be analyzed; and finally, the metabolic characteristics of the heterogeneity of different cell types and their impact on therapeutic responses should be deeply investigated. These breakthroughs in basic research will provide the theoretical foundation and technical guidance needed for developing a new generation of precision nanotherapy strategies.

Additionally, the natural small-molecule compound cryptotanshinone (Cry) could effectively alleviate lactate-induced oxidative stress by modulating the STAT3/SIRT3 signaling pathway (Lu JJ. et al., 2025). Experimental data showed that Cry intervention significantly inhibited the senescence of IVD cells, reduced apoptosis rates, and effectively delayed ECM degradation (Lu JJ. et al., 2025). The highly specific binding of Cry to the STAT3 protein was confirmed through molecular docking and surface plasmon resonance (SPR) techniques. In a rat model of IDD, the Cry-treated group exhibited significant improvement in disease progression (Lu JJ. et al., 2025). These findings provide an experimental basis for treating IDD with natural small-molecule compounds. However, research on small-molecule compounds targeting lactate metabolism and lactylation for IDD remains limited, highlighting an urgent need for the development of small-molecule chemosynthesis strategies.

## 7 Conclusion and future directions

This review synthesizes evidence that lactate and lactylation contribute to the progression of IDD. It classifies inhibitors of lactylation regulation and summarizes the enzymes involved in this metabolic-epigenetic modification. Targeting lactate metabolism has emerged as a promising therapeutic strategy for IDD due to its abundance in degenerating discs, its broad role in cellular processes, and its impact on disc cells. However, despite its potential, most lactate-targeted therapies remain in the preclinical stage, with only a few progressing to clinical trials. Significant challenges remain in the development of effective lactate/lactylation-targeted therapies. One major hurdle is determining the quantitative contribution of lactate modulation to therapeutic efficacy. Additionally, the specificity of the disc structure complicates the achievement of effective drug concentrations, further hindering therapeutic success. Moreover, the true pharmacological mechanisms and therapeutic benefits of current lactate inhibitors *versus* lactate-targeted therapies remain unclear, particularly in relation to the high off-target effects and low specificity to lactate metabolism.

To overcome these challenges, future research should focus on several promising areas to improve therapeutic outcomes. One key area is the design and optimization of novel small-molecule inhibitors that exhibit enhanced specificity and efficacy for NP cells. These inhibitors should selectively disrupt lactate-associated pathways, reducing the metabolic fitness of NP cells. Furthermore, advancements in materials science are needed to develop efficient drug delivery systems that improve bioavailability and precise targeting of therapeutic agents. Such innovations are crucial to ensure that drugs reach their intended site of action in sufficient concentrations while minimizing side effects on healthy tissues. Additionally, greater attention should be given to developing rational and synergistic drug combination strategies. These combination therapies can target multiple pathways simultaneously, maximizing the overall therapeutic effect. On the other hand, future studies on IDD should integrate artificial intelligence (AI) and utilize spatial multi-omics triangulation strategies. For instance, spatial metabolomics can be used to identify and map hotspots of lactate accumulation in pathological regions. Mechanistic decoding can be achieved through joint epigenome-transcriptome profiling to unravel lactate-metabolite regulatory networks. Additionally, spatial transcriptomics and single-cell multi-omics can be applied to map cell types and determine lactate heterogeneity across different cells and microdomains. This synergistic approach will correlate spatiotemporal lactate gradients with lactylation dynamics, reveal cell type-specific metabolic-epigenetic crosstalk, and identify RNA-lactylation interactomes. Ultimately, these efforts will generate spatially resolved maps of IDD pathogenesis, facilitating the development of precision therapies targeting lactate/lactylation pathways.

In conclusion, while lactate-targeted therapies show considerable promise for treating IDD, they remain in the early stages of development. Advancing these therapies will require multidisciplinary collaboration and substantial efforts from researchers across various fields to unlock their full potential. A comprehensive understanding of how lactate interacts with the metabolic and epigenetic processes involved in IDD is crucial for developing more effective therapeutic strategies.
